# Prevalence, Isolation, Identification, and Risk Factors of Major Bacterial Cause of Camel Subclinical Mastitis

**DOI:** 10.1155/2021/5522331

**Published:** 2021-07-01

**Authors:** Atnaf Alebie, Alemante Molla, Wesinew Adugna, Abebe Tesfaye, Mebrat Ejo

**Affiliations:** ^1^School of Veterinary Medicine, Wollo University, P.O. Box 1145, Dessie, Ethiopia; ^2^School of Veterinary Medicine, Welaita Sodo University, P.O. Box 138, Welaita Sodo, Ethiopia; ^3^Microbiology Department, Semera Regional Veterinary Laboratory, P.O. Box 33, Semera, Afar, Ethiopia; ^4^College of Veterinary Medicine and Animal Sciences, University of Gondar, P.O. Box 196, Gondar, Ethiopia

## Abstract

**Objective:**

A crosssectional study was conducted between September 2015 and August 2016 in the district of Afar Regional State, Northeastern Ethiopia, to characterize the most prevalent bacterial pathogens and identify the associated risk factors of camel subclinical mastitis. California mastitis test (CMT) was used as a screening test, and standard bacteriological methods were carried out for isolation and identification of the pathogens.

**Results:**

Among the total 96 lactating camels examined, 25 were found positive with the overall prevalence of 26%, with 25% and 1% subclinical and clinical mastitis cases, respectively. Totally, 384 quarters of udder were examined; of these, 10 of them were blind while the rest 374 were nonblind teats. The quarter level prevalence of subclinical mastitis was 8.9%. The analysis showed that statistically significant difference (*P* < 0.05) of tick infestation and subclinical mastitis. Additionally, among the bacteriologically tested 34 CMT positive milk samples, all of them showed growth on nutrient and blood agar plate. Out of these culture isolates, the major bacterial pathogens identified were *Staphylococcus aureus* (8.7%), *Staphylococcus hyicus* (6.52%)*, Staphylococcus intermedius* (6.52), *Coagulase-negative staphylococci* (19.57%), *Bacillus* (19.57%), *Escherichia coli* (6.52%), and *Pasteurella multocida* (6.52%) species. Therefore, appropriate control measures and awareness creation to the community should be practiced.

## 1. Introduction

The dromedary camel (*Camelus dromedaries*) is a multipurpose animal kept for milk, meat production, and transportation. It is also a financial reserve for pastoralists and plays an important role in social prestige and wealth [1]. Despite its entire significant role, until recently, they were neglected by researchers and development planners in Ethiopia. Research agendas, promotion programs, regular vaccination, and animal health service deliveries are almost always excluding camels. Hence, little is known about their health problems compared to other livestock [2].

Mastitis is a complex disease occurring worldwide among dairy animals with heavy economic losses [3, 4]. It has also multiple hazardous effects on human health. As for other dairy animals, dromedary camel could be affected by mastitis, and subclinical mastitis is more prevalent than clinical mastitis [5].

The causative agents of camel mastitis are not well defined and studied [4]. However, few available literatures indicate that the major bacterial pathogens isolated from subclinical mastitis were *Staphylococcus*, *E. coli*, *Corynebacterium*, *Streptococcus*, *Bacillus*, and *Micrococcus* species [6, 7]. Moreover, according to Abdurahman [8] report, subclinical mastitis is not usually treated in traditionally managed camels and will often take a natural course to chronicity resulting in permanent loss of milk production.

Numerous epidemiological reports have implicated nonheat treated milk and raw-milk products as the major factors responsible for illnesses [9–11], Hence, zoonotic risk arising from this milk should be considered [12]. However, in Afar region in general and in Dubti district in particular, there is scarcity of information on camel mastitis. In the district, greater than 90% of the population is pastoralist, and traditional heat treatment of camel milk is a taboo, so the milk is consumed without any heat treatments. Additionally, the milk is maintained at high ambient temperature after milking and during transportation. Thus, organized problem-oriented research is needed to monitor udder health of camels. Therefore, the objectives of the present study were to estimate the prevalence, identify the major bacterial cause, and assess the effect of risk factors for the occurrence of camel subclinical mastitis.

## 2. Materials and Methods

### 2.1. Study Area and Study Design

A crosssectional study was conducted from September 2015 to June 2016 in Dubti district, Afar Regional State, North-eastern Ethiopia. The area is characterized by high temperature which ranges from 25°C to 42°C. Pastoralism and agro pastoralism are the two major livelihood ways practiced in the area, and there are 147,704 livestock in the district of which 5,966 of them are camels [13]. The study animals were lactating camels which are in various parities (1-10) and lactation stages (l-7 months). Therefore, their lactation stage were grouped in to three categories; 1-3, 4-6, and >6 months as early, mid, and late stage of lactation, respectively, while their parity was categorized as one, two, and >3 births [7].

#### 2.1.1. Sample Size

The sample size was determined as 384 quarter milk sample from 96 camels after calculation by Thrusfield [14] with 95% confidence interval (CI), 5% absolute precision, and 50% expected prevalence.

#### 2.1.2. Milk Sample Collection

During sampling, observation was conducted for the presence of lesion and tick. The samples were collected according to the sterile milk sampling protocol explained by Kirk [15]. First, sterile tube was labeled, and the udder was cleaned and dried using cotton. Then, the end of each teat was sanitized with 70% alcohol starting from the teat that is farthest away to the nearest one and 1-2 streams of milk from each teat were removed. Finally, 75% of the sterile sample tube was filled with the milk samples which are first taken from the nearest one. It was then transported to the laboratory using icebox and placed in a refrigerator at 4°C for less than 72 hours before further processing.

#### 2.1.3. California Mastitis Test (CMT)

California mastitis test was performed before taking milk samples for bacteriology. An equal volume of milk and reagent is mixed, and an evaluation of the degree of gel formation is done after gently rotating CMT paddle. Scores represented four categories: 0, negative (-) or trace (±); 1, positive (+); 2, positive (++); and 3, positive (+++) [16]. Negative (-) and trace (±) reactions were considered as “negative,” and different intensities of positive reactions (+, ++, +++) were considered as “positive” [4].

#### 2.1.4. Bacteriological Culturing and Subculturing

California mastitis test positive milk samples were streaked on blood and nutrient agar plate and incubated for 24-48 hours at 37°C. Then, the plate was read for primary isolation of mastitis pathogens. A single colony from the nutrient agar is also subcultured in nutrient agar/broth medium and incubated for 18 to 24 hour at 37°C [17].

#### 2.1.5. Biochemical Tests

Individual colonies were picked, and their cell morphology and growth on MacConkey agar were observed. Additionally, Gram stain, catalase, oxidase, oxidation fermentation (OF), and motility tests were conducted to identify the genera of bacterial species [17, 18]. Then, coagulase and Triple Sugar Iron (TSI) test were done to identify *coagulase-positive staphylococcal* species and *Enterobacteriaceae* family, respectively. Moreover, haemolysis on blood agar plate, its pigment production, and growth on Manitol, Maltose, and Trehalose broth media were observed to identify *Staphylococcus* species [17, 19]. Indol, Metyl-Red (MR), Voges-Proskauer (VP), citrate utilization, lysine decarboxylase, and urease test were also done for identification of *Enterobacteriaceae* and *Pasturella* spp. [20]. The positive controls for each biochemical tests are known bacteria which are positive for every test.

#### 2.1.6. Statistical Analysis

The data was fed into MS-Excel spread sheets and analyzed using STATA (MP16.0). The association of subclinical mastitis with parity, stage of lactation, tick infestation, lesion, kebele, and herd size were compared using chi-square test (*χ*^2^). Furthermore, logistic regression analysis was performed to quantify odds ratio, and *P* < 0.05 is considered statistically significant.

## 3. Results and Discussion

The result of the present study indicated that subclinical mastitis is widespread with an overall prevalence rate of 25% at she-camel and 8.85% at quarter level. This result at she-camel level is lower than that reported by Regassa et al. [21] and Suheir et al. [22] who found an overall prevalence of 39.4% and 36.87% in Ethiopia and Sudan camel herds, respectively. This variation could be due to the fact that environmental factors play significant role in the prevalence of subclinical mastitis [4]. Another possible reason could be in the study area, some of the factors which can predispose camel udders to bacterial infections, i.e., the practice of camel herders cauterizing the udder so as to treat mastitis and putting sticks into the nostrils of calves to prevent suckling reported by Mengistu et al. [7] is not practiced. Moreover, the unhygienic milking procedure and generally poor management practice might also have contributed to the higher prevalence of mastitis in the camel herds examined by those previous researchers.

In this study, the association of subclinical mastitis with different influencing factors, such as tick infestation, lesion, parity, stage of lactation, and herd size, was assessed for any possible correlation (Table 1). Among them, the high prevalence of subclinical mastitis in tick infested she-camels (52%) is observed, and tick infested she-camels were 5.91 times more susceptible than nontick infested she-camels. The variation in prevalence was also statistically significant (*P* < 0.05). The possible reason for this could be due to the fact that tick infestation can predispose camel udders to bacterial infection [23], which is because of tick bites on the udder can cause skin irritation and localized inflammatory response that can lead to secondary bacterial infections [7]. Though other possible risk factors were not statistically significant, there was a variation between their prevalence. The high prevalence (9.38%) of udder/teat lesion is found in the present study area. This may be due to scratches caused by thorny plants of the desert or it could be attributed to the tick burden infesting the udders. The nonsignificant association (*P* > 0.05) with subclinical mastitis may be due to the lesion that was mostly nonpenetrating superficial wound. Thus, the chance of microorganisms penetrating in to the udder through this wound is not much high.

Additionally, even if there is no statistically significant variation in the prevalence of subclinical mastitis with respect to stage of lactation, there is variation among different stages. It is high in early (23.33%) and midstage of lactation (27.42) and low in the last stage of lactation which is in line with the finding of Mengistu et al. [7] and Regassa et al. [21] who reported high prevalence of subclinical mastitis in early stage of lactation. The highest prevalence in midstage of lactation may be due to the fact that, in the study area, she-camels are not usually milked for the first two to three weeks after they give birth. Hence, this might decrease the degree of contamination of the udder. However, according to Suheir et al. [22], few cases of mastitis were observed (25%) at the first stage, (30%) at the second stage, and higher number of cases at the last stage of lactation (45%).

Variation is also observed among she-camels in different parities; in animals at their first calving, the occurrence of subclinical mastitis was 50%, while in camels that gave two births, the rate decreased to 28.57% but sharply raised to 77.63% in she-camels that gave three or more births (Table 1). This result is in line with the finding of Mengistu et al. [7] who reported that subclinical mastitis was prevalent in she-camels with three or more parity. However, the present study disagrees with the finding of Suhier et al. [22] who reported that during the first, second, and third calving, the prevalence of mastitis was 25% which was increased to 43.8% at the fourth and fifth calving, while it is decreased in to 16.7% in the sixth, seventh, and eighth calving. The cause of increasing subclinical mastitis with parity could be linked to a less immunity defense, a change in udder morphology (higher elasticity of mammary gland), and increasing of udder trauma with the number of parities [24].

Moreover, an equal proportion of animals from each kebele was found positive for subclinical mastitis which had not significant variation (Table 1). This can be due to the fact that even if there is territorial demarcation between them, the camel owners are pastoralists; hence, they move their camels from one kebele to the other kebele, and also, there is no any difference in environment and management system between them. Similarly, there was no statistically significant difference in the prevalence rate of camel subclinical mastitis in camel herd sizes. However, variation in prevalence rate exists among them, the highest being at herd size greater than twenty (30%) followed by in herd size between one to ten (25%) and least in herd size between eleven to twenty (21.4%) (Table 1). This may be due to individual difference in hygienic milking practice among the herds as well as the higher herd size may lead the owners for negligence of hygienic milking practice.

**Figure 1:**
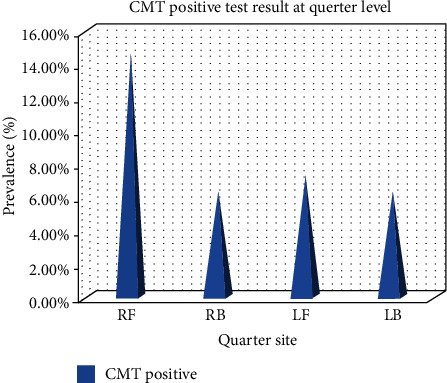
Occurrence of camel subclinical mastitis in different quarters.

Furthermore, the variation in the occurrence of subclinical mastitis among the four quarters was assessed, and it showed that the right front (RF) quarter (14.6%) is the most affected quarters, followed by 7.3% left front (LF), and 7.3% right hind (RH) and 6.3% left hind (LH) (Figure 1). This may be due to the anatomy of camel udder with a narrow basin that could explain a better protection of the hind quarters compared to the front ones [24]. On the other hand, the trend that most of the camel milkers in the study area usually start milking from the right front quarter which may increase the chance of direct microbial contamination.

It is known that milk is a good medium for several bacteria to develop [10]. Hence, isolation and identification of bacteria was also done to determine which bacteria are present in camel milk and to what extent. Out of 384 quarters examined, a total of 374 quarter milk samples were collected and used for analysis using CMT because ten (2.6%) were blind teats. Of these milk samples, 34 (8.85%) were CMT positive for subclinical mastitis, and all of them yielded bacteria up on culturing. As depicted in Table 2, 46 isolates of different bacteria were recorded, such as *S. aureus*, *S. hyicus*, *S. intermedius*, *coagulase-negative staphylococcus* spp., *Bacillus* spp., *E. coli*, *P. multocida*, *P. haemolytica*, *Nisseria* spp., *Micrococcus*, *Aeromonas*, and *Acinobacter* species.

The prevalence of *Staphylococci* spp. varies according to the different studies, but there is nearly no investigation on the bacteriological hygiene of camel milk where *staphylococci* are not mentioned [10]. In several investigations on milk of healthy camels, *coagulase-positive staphylococcus* (CPS) results were contradictory, and their prevalence is given with 0.5-24.7% [25] which agrees with the present finding (21.74%). Among them, *S. aureus* was mentioned the main cause of subclinical camel mastitis [10]. In this study, 8.7% of *S. aureus* is found much higher than the finding by Almaw and Molla [26] who reported 0.6% but lower than Woubit et al. [27] and Mengistu et al. [7] who informed 21.03% and 16%, respectively. Additionally, *S. hyicus* (6.52%) is higher than the result by Suheir et al. [22] who reported 2.63%, 1.32%, and 3.95% in Kordofan, Portsudan, and Kartoum, respectively. However, both *S. hyicus* and *S. intermedius* (6.52% each) are much lower than reported by Woubit et al. [27] who found 25.34% and 82.41%, respectively. Such high prevalence of CPS in the present study in addition to traditional taboo on heat treatment of camel milk and maintaining milk at high ambient temperature after milking and during transportation in the study area can pose a serious problem to human health as these practices create conducive situation for the production of staphylococcal enterotoxin [28]. Out of the total isolates, 19.57% of *coagulase-negative staphylococci* spp. (CNS) was also isolated from most of the CMT positive milk samples which agrees with the finding of Woubit et al. [27] (18.2%). However, it is lower than Mengistu et al. [7] who reported 40.4%. Though it is reported that these *Staphylococci* spp. are known as facultative (“minor”) pathogens isolated from subclinical mastitis cases which do not show a measurable influence on milk yield, CMT, or clinical symptoms [10], an explanation for their frequent occurrence is most probably due to the contamination of the milk samples by the teat canal or teat skin.

*Bacillus* spp. were found in 19.57% of the total isolates which is higher than Mengistu et al. [7] and Woubit *et al*. [27] who reported that 4.3% and 10.82%, respectively. The higher prevalence of *Bacillus* spp. reported in the present study could be due to poor milking hygiene and contamination from soil.

*E. coli* (6.52%) and *Klebsiella pnumoniae* (4.35%) were also isolated in variable numbers. This result is in agreement with the findings of Mengistu et al. [7] which is 9.6% and 2.1%, respectively. As coliforms can be a sign of insufficient hygienic conditions and to a minor degree of faecal contamination [10], the prevalence may vary considerably according to hygiene conditions.

Furthermore, *pasturella* spp. are found the cause of subclinical mastitis. The prevalence of *P. haemolityica* (4.35%) in the present study is comparable with those reported by Mengistu et al. [7], Gadir et al. [29], and Semereab and Molla, [30] 3.2%, 2.1%, and 5.4%, respectively, but higher than Woubit et al. [27] who reported 0.12%. *P. multocida* (6.12%) and *Mannheimia haemolytica* (4.35%) isolated in this study from subclinical cases were also isolated from clinical camel mastitis by Fazlani et al. [31] which proved their potentiality to cause mastitis.

The species of *Aeromonas, Neisseria*, and *Acinobacter* have been also isolated with a low prevalence. It is reported that *Aeromonas* spp. are widely spread in fresh water, sewage, and soil. It can occasionally cause infections in humans that range from wound to self-limiting diarrhea and animals can be carriers of these species. However, bacteria like *Neisseria* and *Acinobacter* spp. have minor veterinary importance and can be found in soil, water, sewage, food, and milk [17]. *Micrococcus* spp. is also isolated in this study as 4.35% of the total isolates which is in line with Mengistu et al. [7], Saleh and Faye [24], and Woubit et al. [27] who reported 6.4%, 5.7%, and 10.58%, respectively.

Generally, it is known that failure to maintain adequate sanitation practices contributes to contamination of milk with undesirable or pathogenic microorganisms [32]. The common predisposing factors for contamination of milk are the milking environment, the milk handling personnel, and the cow (udder). Microorganisms can be transferred from the environment, i.e., faeces, bedding, and soil; from contaminated hands, clothing and mouth of milk handling personnel; and from dirty water and clothes used for cleaning udder to the exterior of the cows' udder and teats. Then, those microorganisms that are attached to the exterior of the teats/udder can enter the teat canal and increase the risk of occurrence of mastitis [33]. Therefore, identification of these bacteria in the present study may be due to contamination of the camels' udder by the hands of unhygienic milkers or unhygienic milking procedure. However, though the prevalence of subclinical mastitis can be affected by hygienic and other management practices, it is not considered in this study. Thus, failure to include these factors should be considered as the limitation of the study.

## 4. Conclusions

In conclusion, the results of the present study showed that subclinical mastitis was prevalent, and it was majorly attributed to tick infestation. Those pathogens isolated are bacteria that cause both environmental and contagious mastitis which suggest that there is lack of proper management and adequate hygienic condition. Thus, any endeavor towards animal disease control strategy must include camel subclinical mastitis among the priority list. Tick control measures and health education are aimed at increasing awareness of the people about camel subclinical mastitis; the importance of good management practices with proper sanitation during the production and handling of camel milk and the benefits accompanied of its control is essential.

## Figures and Tables

**Table 1:** Association of different risk factors with camel subclinical mastitis in Dubti district from September 2015 to August 2016.

Risk factors	No. of she-camels examined	CMT			
		Number positive, *n* (%)	OR [95% CI]	*P* value	*χ* ^2^
*Tick infestation*					
Positive	25	13 (52)	5.91 [2.14-16.29]	0.001	13.143
Negative	71	11 (15.49)			
*Lesion*					
Positive	9	3 (33.4)	1.57 [0.36-0.19]	0.55	0.37
Negative	87	21 (24.13)			
*Kebele*					
Dubti	32	8 (25)	1 [0.38-2.66]	1	0.00
Logia	64	16 (25)			
*Stage of lactation*					
Early	30	7 (23.33)	0.8 [0.35-1.85]	0.6	1.43
Mid	62	17 (27.42)			
Late	4	0 (0)			
*Parity*					
One	6	3 (50)	0.56 [0.28-1.13]	0.11	2.82
Two	14	4 (28.57)			
>3 births	76	59 (77.63)			
*Herd size*					
1-10	24	6 (25)	1.16 [0.62-2.16]	0.64	0.69
11-20	42	9 (21.4)			
>21	30	9 (30)			

**Table 2:** Distribution of isolates and individual prevalence of bacterial species isolated from camels in the district of Dubti.

Bacteria isolated	No. of isolates	% of isolates
*Staphylococcus aureus*	4	8.7
*Staphylococcus hyicus*	3	6.52
*Staphylococcus intermedius*	3	6.52
*Coagulase-negative staphylococcus*	9	19.57
*Escherichia coli*	3	6.52
*Klebsiella pneumoniae*	2	4.35
*Pasteurella multocida*	3	6.52
*Mannheimia haemolytica*	2	4.35
*Micrococcus* spp.	2	4.35
*Acinobacter* spp.	1	2.17
*Nisseria* spp.	4	8.7
*Aeromonas* spp.	1	2.17
*Bacillus* spp.	9	19.57
Total	46	100

## Data Availability

All relevant data are contained within the article.
